# Integrative analysis reveals methylenetetrahydrofolate dehydrogenase 1-like as an independent shared diagnostic and prognostic biomarker in five different human cancers

**DOI:** 10.1042/BSR20211783

**Published:** 2022-01-06

**Authors:** Nuzhat Sial, Jalil Ur Rehman, Saba Saeed, Mukhtiar Ahmad, Yasir Hameed, Muhammad Atif, Abdul Rehman, Rizwan Asif, Hamad Ahmed, Muhammad Safdar Hussain, Muhammad Rashid Khan, Atifa Ambreen, Ayesha Ambreen

**Affiliations:** 1 Department of Zoology, The Islamia University of Bahawalpur, Bahawalpur, Pakistan; 2 Department of Eastern Medicine, Qarshi University, Lahore, Pakistan; 3 University College of Conventional Medicine, The Islamia University of Bahawalpur, Bahawalpur, Pakistan; 4 Department of Zoology, University of the Punjab, Lahore, Pakistan; 5 Department of Biochemistry and Biotechnology, The Islamia University of Bahawalpur, Bahawalpur, Pakistan; 6 Department of Eastern Medicine, Qarshi University, Lahore, Pakistan; 7 Department of Microbiology, Government College University Faisalabad, Faisalabad, Pakistan; 8 Department of Eastern Medicine, Government College University Faisalabad, Faisalabad, Pakistan; 9 University College of Eastern Medicine, The Islamia University of Bahawalpur, Bahawalpur, Pakistan; 10 Allied Department, The Sahara College, Narowal, Pakistan

**Keywords:** Biomarker, Cancer, Expression variations, MTHFD1L

## Abstract

**Background:** Defects in methylenetetrahydrofolate dehydrogenase 1-like (MTHFD1L) expression have earlier been examined in only a few human cancers. **Objectives:** Multi-omics profiling of MTHFD1L as a shared biomarker in distinct subtypes of human cancers. **Methods:** In the current study, for the multi-omics analysis of MTHFD1L in 24 major subtypes of human cancers, a comprehensive *in silico* approach was adopted to mine different open access online databases including UALCAN, Kaplan–Meier (KM) plotter, LOGpc, GEPIA, Human Protein Atlas (HPA), Gene Expression across Normal and Tumor tissue (GENT2), MEXPRESS, cBioportal, STRING, DAVID, TIMER, and Comparative Toxicogenomics Database (CTD). **Results:** We noticed that the expression of MTHFD1L was significantly higher in all the analyzed 24 subtypes of human cancers as compared with the normal controls. Moreover, MTHDF1L overexpression was also found to be significantly associated with the reduced overall survival (OS) duration of Bladder urothelial cancer (BLCA), Head and neck cancer (HNSC), Kidney renal papillary cell carcinoma (KIRP), Lung adenocarcinoma (LUAD), and Uterine corpus endometrial carcinoma (UCEC). This implies that MTHFD1L plays a significant role in the development and progression of these cancers. We further noticed that MTHFD1L was also overexpressed in BLCA, HNSC, KIRP, LUAD, and UCEC patients of different clinicopathological features. Pathways enrichment analysis revealed the involvement of MTHFD1L-associated genes in five diverse pathways. We also explored few interesting correlations between MTHFD1L expression and its promoter methylation, genetic alterations, CNVs, and between CD8+ T immune cells level. **Conclusion:** In conclusion, our results elucidated that MTHFD1L can serve as a shared diagnostic and prognostic biomarker in BLCA, HNSC, KIRP, LUAD, and UCEC patients of different clinicopathological features.

## Introduction

Cancer is the second most common cause of mortality behind cardiovascular diseases worldwide [1]. Despite improvements in cancer detection and treatment methods, cancer incidence continues to rise rapidly, resulting in huge economic losses and premature deaths throughout the world [2]. In accordance with the World Health Organization (WHO) Cancer Stats 2020, approximately 18 million new cancer cases and 9.6 million deaths were estimated around the globe [3].

Many well-known cancer-causing risk factors include tobacco smoking, alcohol consumption, hepatitis B or C infection, diabetes, and obesity [2,4,5]. When someone tests positive for cancer, its prognosis mainly depends on the stage of the tumor at the time of detection. The early diagnosis of cancer is useful to obtain the more appropriate treatment options [6]. However, prior to our knowledge, there is a dearth of effective cancer-associated biomarkers that could commonly use to detect multiple cancer subtypes together as a shared biomarker and aid in the targeted therapy.

Metabolic disorders are one of the key cancer hallmarks because of their involvement in tumor growth and invasion [7]. Mainly, cancerous cells depend upon the folate cycle to grow further. The folate cycle is responsible for fulfilling numerous cancer-associated nutritional requirements. A cytoplasmic enzyme, known as Methylenetetrahydrofolate dehydrogenase 1-like (MTHFD1L) participates in the formation of tetrahydrofolate (THF) within mitochondria [8], which is further involved in the folic acid cycle to synthesize formate [9]. Folic acid deficiency can lead to a variety of diseases including immune system dysfunction and cancer [10]. In 1949, Farber and Farber were the first to observe the tumor-associated role of folic acid in leukemic cells [11]. Their keen observation of folate’s cancer-associated role has laid the foundation of chemotherapeutic agents development as a single or in combination with treatment options for cancer patients [11]. A recent report revealed that MTHFD1L plays a vital role in bladder cancer by increasing cell proliferation, invasion and metastasis [12], however, their results were not validated further. Another study has also reported the role of MTHFD1L in colorectal cancer (CRC) development and progression. In view of the results of this study, MTHFD1L expression blocking reduces the CRC cells growth and development, thus providing MTHFD1L as a new avenue in CRC treatment to be targeted with different inhibitors [9]. Furthermore, MTHFD1L was also found to be associated with esophageal squamous cell carcinoma (ESCA) [13] and tongue squamous cell carcinoma [14]. Despite the significance of MTHFD1L in bladder cancer, CRC, ESCA and squamous cell carcinoma, knowledge regarding its role in other subtypes of human cancer is still unknown.

In the present work, to uncover the MTHFD1L expression variations across different cancers, we analyzed a large tumor sample size paired with controls from different reliable online databases through a comprehensive bioinformatics approach. The findings of the present study have helped us to evaluate the crucial role of MTHFD1L as a shared biomarker in predicting the prognosis, performing the diagnosis, and initiating the development of Bladder urothelial cancer (BLCA), Head and neck cancer (HNSC), Kidney renal papillary cell carcinoma (KIRP), Lung adenocarcinoma (LUAD), and Uterine corpus endometrial carcinoma (UCEC).

## Methods

### UALCAN

UALCAN (http://ualcan.path.uab.edu) is an effective online tool for multi-omics data analysis of any gene(s) of interest across 31 cancer subtypes. This tool obtained multi-omics data from the TCGA projects [15] and also provide correlations between gene expression and different relevant clinicopathological features. In our study, this tool was selected to determine the differential expression of MTHFD1L in various cancer subtypes relative to normal controls. For this purpose, we initially entered MTHFDL1 gene name in the search box of UALCAN, then chose the pan-cancer analysis option to obtain the graph illustrating MTHFDL1 expression across multiple cancers in the form of box plots. In the obtained graph, the transcription expression level of MTHFD1L was quantified as transcript per million (TPM) reads, and a cutoff of *P*-value was selected as <0.05 in the Student’s *t* test.

### Kaplan–Meier plotter, GEPIA, UALCAN, and LOGpc tools

The prognostic values (OS duration) of MTHFD1L across different cancers was analyzed via Kaplan–Meier (KM) plotter (https://kmplot.com/analysis/) and GEPIA (http://gepia.cancer-pku.cn/) tools [16,17]. Moreover, UALCAN (http://ualcan.path.uab.edu) [15] was used to evaluate MTHFD1L prognostic values in cancer patients of different stages, races and genders, while LOGpc (https://bio.tools) [18] has helped to explore MTHFD1L prognostic values in cancer patients of different age groups. To do so, we entered the name of the MTHFDL1 gene into the search boxes of each tool, then adjusted the survival analysis type to OS and clinical parameters to cancer stage, race, gender in case of UALCAN, while age in the case of LOGpc. We then plotted the KM curves using default settings. During the analysis, cancer specimens were categorized into two groups by median expression level (high v/s low expression level) and a *P*-value was selected as <0.05.

### Gene Expression across Normal and Tumor tissue database

For further validating the MTHFD1L transcription expression in tumor tissues, we analyzed NCBI GEO datasets of independent cohorts of distinct cancer patients using Gene Expression across Normal and Tumor tissue (GENT2) database with default settings (http://gent2.appex.kr/) [19]. In the analysis, a cutoff of *P*-value was selected as <0.05 in the Student’s *t* test.

### Data mining through Human Protein Atlas database

The Human Protein Atlas (HPA) database (http://www.proteinatlas.org) provides immunohistochemistry (IHC)-based proteomics expression data for any gene(s) of interest across more than 20 major subtypes of human cancers [20]. Via this database, researchers can easily identify the differentially expressed protein in the tissues of a specific cancer relative to controls. In this work, the IHC images of MTHFD1L proteomics expression in distinct human cancer tissues relative to normal tissues were visualized via HPA. The observed protein expression level was graded as not detected, low, medium and high, based on the intensity of staining and fraction of the stained cells. A *P*-value of <0.05 was chosen as significant.

### MEXPRESS

MEXPRESS (https://mexpress.be/) web tool is designed for TCGA gene expression, promoter methylation, and a Pearson correlation analysis between these parameters for any gene(s) of interest in distinct cancer subtypes [21]. In our study, we used this tool to find out the correlation among expression and promoter methylation level of MTHFD1L in distinct cancer subtypes with default settings. A cutoff of *P*-value was selected as <0.05 in the Pearson correlation test.

### The cBioportal database

The cBio000Portal (http://cbioportal.org) open-access database is dedicated to analyzing multi-omics cancer data from TCGA projects, which collectively includes more than 715 datasets and 86733 cancer samples [22]. In this study, different TCGA PanCancer Atlas datasets were chosen to examine MTHFD1L genomic alterations (mutations and copy number variations) across distinct human cancer subtypes. The search terms included mutations and putative copy number for MTHFD1L. The OncoPrint tab on the cBioPortal results interface has provided the overview of genetic mutations and CNVs above every sample bar.

### Predicted protein–protein interaction network and pathways of MTHFD1L

In the current study, STRING tool (https://string-db.org/cgi/input?sessionId=befKymonvu2t&input_page_show_search=on) [23], which is dedicated to predicting protein–protein interactions (PPIs) of any gene(s) of interest, was used to obtain the PPI network of MTHFD1L-associated genes with default settings. Later, for the pathway enrichment of MTHFD1L-enriched genes, DAVID (http://david.ncifcrf.gov/summary.jsp) [24] was utilized with the default settings. A *P*-value of <0.05 was considered as significant in the analysis.

### Infiltrating level of CD8+ T in relationship with MTHFD1L across different human cancers

TIMER database (https://cistrome.shinyapps.io/timer/) systematically used microarray-based expression values for computing a Pearson correlation between immune cell infiltrates and the expression level of any gene(s) of interest across diverse cancer subtypes from TCGA data (10897 samples across 32 cancer types) [25]. In our study, CD8+ T immune cell infiltrates of MTHFD1L in different cancers were calculated using TIMER, and a *P*-value of <0.05 was considered as significant in the Pearson correlation test.

### Chemotherapeutic drugs associated with MTHFD1L

MTHFD1L-associated chemotherapeutic drugs were obtained from the Comparative Toxicogenomics Database (CTD) [26] database for constructing a gene–drug interaction network via Cytoscape. This database can also be used to analyze gene–chemistry, gene–disease, and chemical–disease interaction networks. In our study, a constructed MTHFD1L gene–drug interaction network includes information on chemotherapeutic drugs that can reduce or enhance the expression level of MTHFD1L.

## Results

### MTHFD1L expression in human cancers and normal tissues

**Figure 1:**
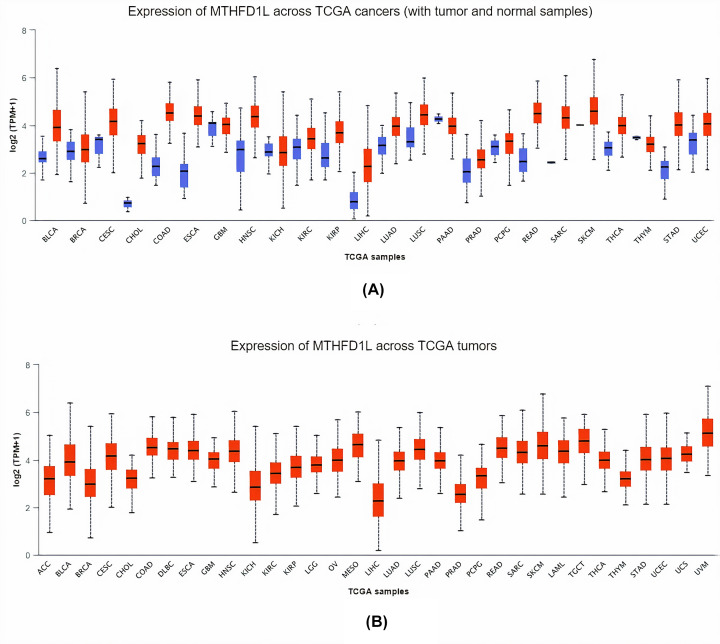
The analysis of difference in transcription level of MTHFD1L in different human cancer tissues paired with normal controls (**A**) Transcription level analysis of MTHFD1L across cancerous samples paired with normal controls, and (**B**) transcription level analysis of MTHFD1L across cancerous samples only without relating to normal controls. Blue color represents the normal samples, while red color indicates the cancer samples. A *P*-value (<0.05) was considered as statistically significant.

To explore the MTHFD1L mRNA expression levels in 24 major subtypes of human cancer tissues paired with normal control, TCGA expression data were analyzed using pan-cancer analysis via UALCAN platform. Results of the analysis showed a significant (*P*<0.05) up-regulation of MTHFD1L in all 24 major cancer tissues relative to controls (Figure 1).

### The prognostic values of MTHFD1L in human cancers calculated via KM plotter and GEPIA

**Figure 2:**
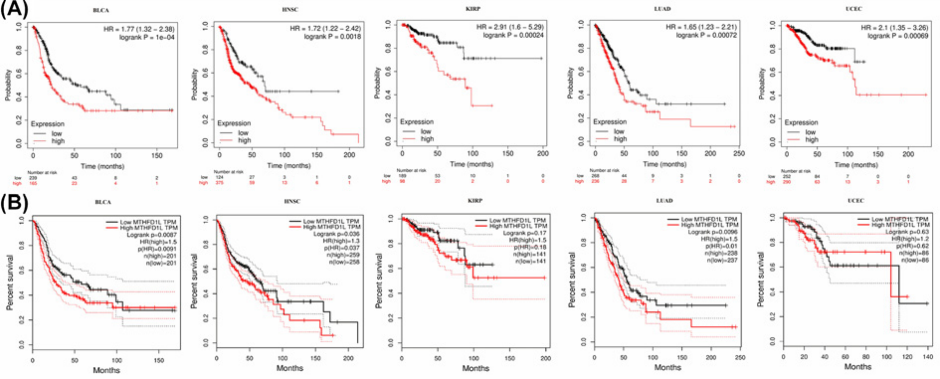
UALCAN and GEPIA-based correlation analysis of MTHFD1L overexpression with the OS of distinct cancer patients (**A**) UALCAN-based correlation analysis of MTHFD1L overexpression with the OS of BLCA, HNSC, KIRP, LUAD, and UCEC patients, and (**B**) GEPIA-based correlation analysis of MTHFD1L overexpression with the OS of BLCA, HNSC, KIRP, LUAD, and UCEC patients. A *P*-value (<0.05) was considered as statistically significant.

Next, to check whether MTHFD1L higher expression has any effect on the overall survival (OS) duration of the 24 types of cancer patients or not, we used KM plotter and GEPIA tools for OS analysis. In view of both resources collective results, a higher MTHFD1L expression was found to be associated with the reduced OS duration of the BLCA, ESCA, HNSC, KIRP, LUAD, and UCEC patients only and not with any other subtype (Figure 2). Altogether, our data suggested that MTHFD1L might have a significant contribution to the development and progression of BLCA, HNSC, KIRP, LUAD, and UCEC, thus the next part of our study will mainly focus on the unique role of MTHFD1L in these five types of human cancers.

### Clinicopathological feature-specific mRNA expression of MTHFD1L

**Figure 3:**
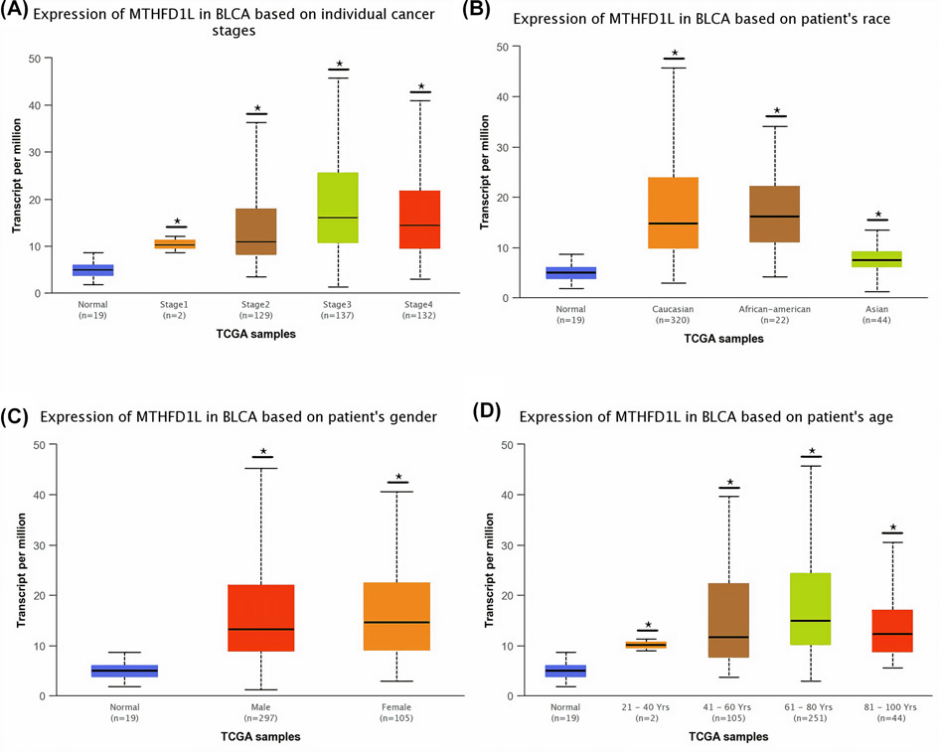
The relationship of MTHFD1L expression with different clinicopathological features of BLCA (**A**) Expression of MTHFD1L in different cancer stages of BLCA patients, (**B**) expression of MTHFD1L in different races of BLCA patients, (**C**) expression of MTHFD1L in different genders of BLCA patients, and (**D**) expression of MTHFD1L in different age groups of BLCA patients. *P<0.05.

**Figure 4:**
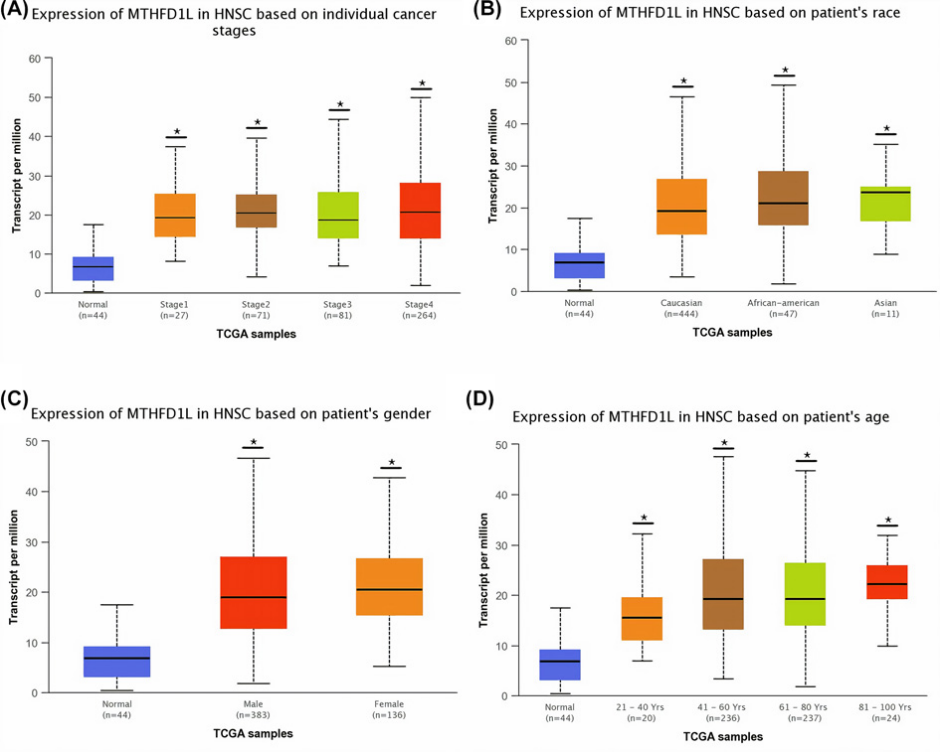
The relationship of MTHFD1L expression with different clinicopathological features of HNSC (**A**) Expression of MTHFD1L in different cancer stages of HNSC patients, (**B**) expression of MTHFD1L in different races of HNSC patients, (**C**) expression of MTHFD1L in different genders of HNSC patients, and (**D**) expression of MTHFD1L in different age groups of HNSC patients. *P<0.05.

**Figure 5:**
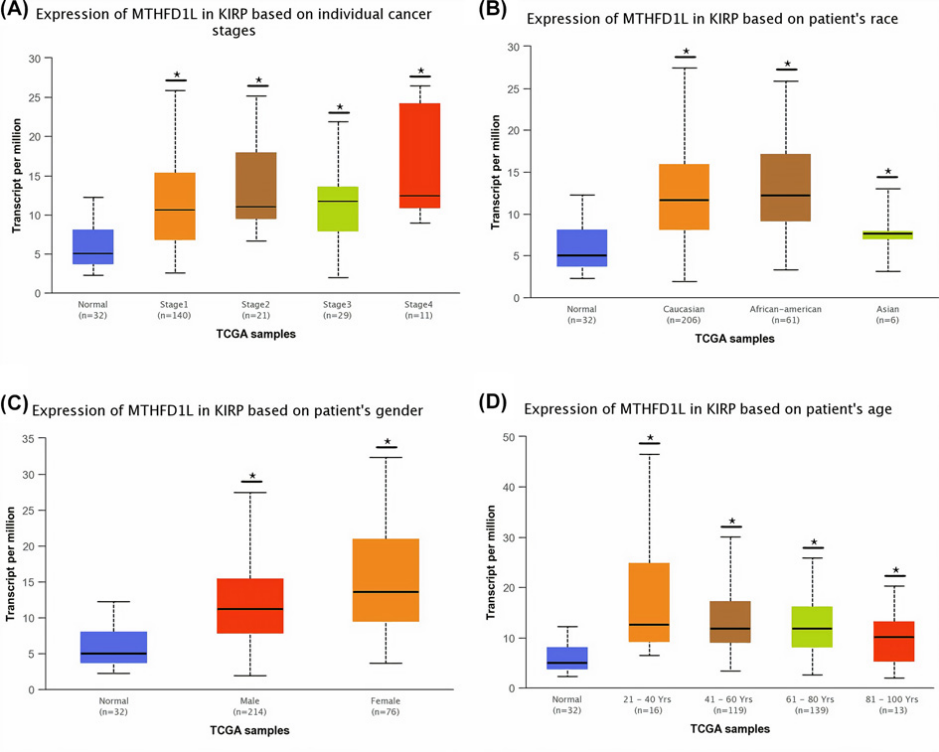
The relationship of MTHFD1L expression with different clinicopathological features of KIRP (**A**) Expression of MTHFD1L in different cancer stages of KIRP patients, (**B**) expression of MTHFD1L in different races of KIRP patients, (**C**) expression of MTHFD1L in different genders of KIRP patients, and (**D**) expression of MTHFD1L in different age groups of KIRP patients. *P<0.05.

**Figure 6:**
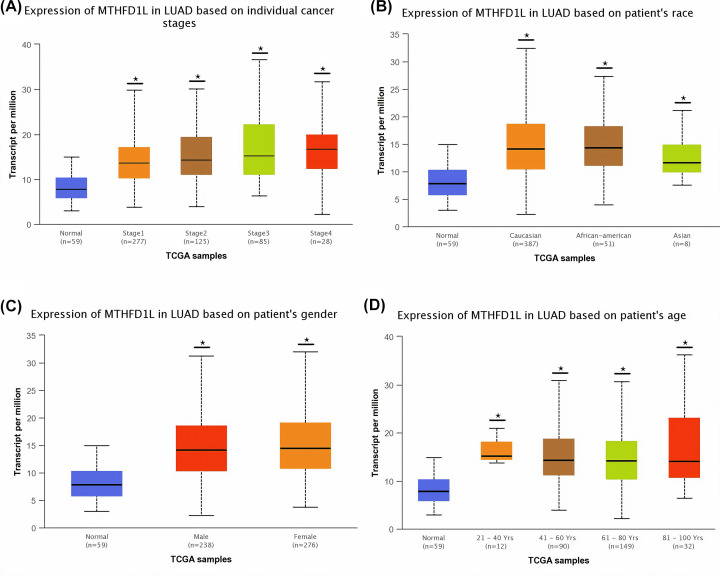
The relationship of MTHFD1L expression with different clinicopathological features of LUAD (**A**) Expression of MTHFD1L in different cancer stages of LUAD patients, (**B**) expression of MTHFD1L in different races of LUAD patients, (**C**) expression of MTHFD1L in different genders of LUAD patients, and (**D**) expression of MTHFD1L in different age groups of LUAD patients. *P<0.05.

**Figure 7:**
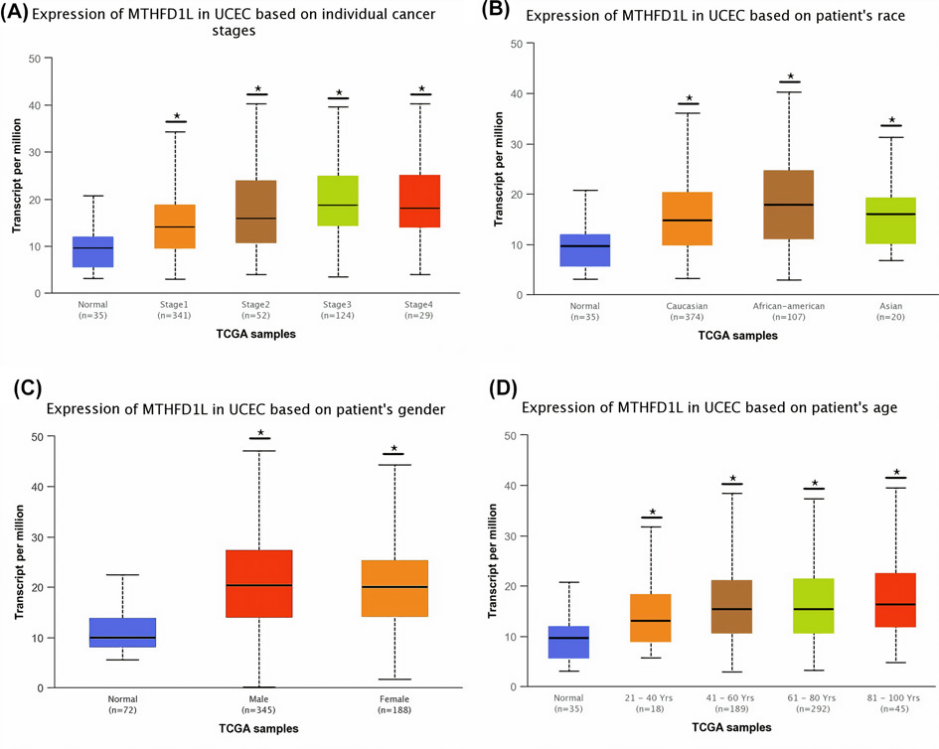
The relationship of MTHFD1L expression with different clinicopathological features of UCEC (**A**) Expression of MTHFD1L in different cancer stages of UCEC patients, (**B**) expression of MTHFD1L in different races of UCEC patients, (**C**) expression of MTHFD1L in different genders of UCEC patients, and (**D**) expression of MTHFD1L in different age groups of UCEC patients. *P<0.05.

To detect the clinicopathological feature-specific expression of MTHFD1L in BLCA, HNSC, KIRP, LUAD, and UCEC, we used the UALCAN platform. In view of the results of our analysis, a significant (*P*<0.05) overexpression of MTHFD1L was also observed in BLCA, HNSC, KIRP, LUAD, and UCEC patients of different clinicopathological features including different cancer stages, race, gender, and age (Figures 3-7). A clinical parameter-wise classification of the BLCA, HNSC, KIRP, LUAD, and UCEC cohorts can be seen in Tables 1-3.

**Table 1:** Details of the clinicopathological feature-based classification of BLCA and HNSC samples analyzed in the current study

Sr. No.	Clinicopathological feature	Sample count	Total sample count	Sample count with missing information	Total final sample count undertaken in analysis
**Clinicopathological feature-based classification of BLCA samples**					
1	**Cancer stage distribution**		408		
	Stage 1	02			
	Stage 2	129		08	400
	Stage 3	137			
	Stage 4	132			
2	**Geographical distribution**				
	Caucasian	320			
	African-American	22		22	386
	Asian	44			
3	**Gender distribution**				
	Male	297		06	402
	Female	105			
4	**Age distribution**				
	21–40 years	02			
	41–60 years	105		06	402
	61–80 years	251			
	81–100 years	44			
**Clinicopathological feature-based classification of HNSC samples**					
1	**Cancer stage distribution**		520		
	Stage 1	27			
	Stage 2	71		77	443
	Stage 3	81			
	Stage 4	264			
2	**Geographical distribution**				
	Caucasian	444			
	African-American	47		18	502
	Asian	11			
3	**Gender distribution**				
	Male	383		01	519
	Female	136			
4	**Age distribution**				
	21–40 years	20			
	41–60 years	236		03	517
	61–80 years	237			
	81–100 years	24			

**Table 2:** Details of the clinicopathological feature-based classification of KIRP and LUAD samples analyzed in the current study

Sr. No.	Clinicopathological feature	Sample count	Total sample count	Sample count with missing information	Total final sample count undertaken in analysis
**Clinicopathological feature-based classification of KIRP samples**					
1	**Cancer stage distribution**		290		
	Stage 1	140			
	Stage 2	21		89	201
	Stage 3	29			
	Stage 4	11			
2	**Geographical distribution**				
	Caucasian	206			
	African-American	61		17	273
	Asian	06			
3	**Gender distribution**				
	Male	214		00	290
	Female	76			
4	**Age distribution**				
	21–40 years	16			
	41–60 years	119		03	287
	61–80 years	139			
	81–100 years	13			
**Clinicopathological feature-based classification of LUAD samples**					
1	**Cancer stage distribution**		515		
	Stage 1	277			
	Stage 2	125		0	515
	Stage 3	96			
	Stage 4	28			
2	**Geographical distribution**				
	Caucasian	387			
	African-American	51		69	446
	Asian	08			
3	**Gender distribution**				
	Male	238		01	514
	Female	276			
4	**Age distribution**				
	21–40 years	12			
	41–60 years	90		277	283
	61–80 years	149			
	81–100 years	32			

**Table 3:** Details of the clinicopathological feature-based classification of UCEC samples analyzed in the current study

Sr. No.	Clinicopathological feature	Sample count	Total sample count	Sample count with missing information	Total final sample count undertaken in analysis
**Clinicopathological feature-based classification of UCEC samples**					
1	**Cancer stage distribution**		546		
	Stage 1	341			
	Stage 2	52		0	546
	Stage 3	124			
	Stage 4	29			
2	**Geographical distribution**				
	Caucasian	374			
	African-American	107		45	501
	Asian	20			
3	**Gender distribution**				
	Male	348		10	536
	Female	188			
4	**Age distribution**				
	21–40 years	18			
	41–60 years	189		02	544
	61–80 years	292			
	81–100 years	45			

### MTHFD1L has good prognostic significance in BLCA, HNSC, KIRP, LUAD, and UCEC patients regardless of different clinicopathological features

**Figure 8:**
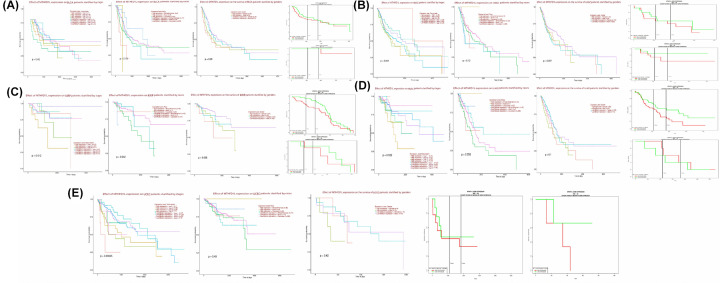
UALCAN and LOGpc-based prognostic significance of MTHFD1L overexpression in BLCA, HNSC, KIRP, LUAD, and UCEC patients of different clinicopathological features (**A**) UALCAN and LOGpc-based MTHFD1L prognostic significance in BLCA patients of different cancer stages, races, genders, and ages. (**B**) UALCAN and LOGpc-based MTHFD1L prognostic significance in HNSC patients of different cancer stages, races, genders, and ages. (**C**) UALCAN and LOGpc-based MTHFD1L prognostic significance in KIRP patients of different cancer stages, races, genders, and ages. (**D**) UALCAN and LOGpc-based MTHFD1L prognostic significance in LUAD patients of different cancer stages, races, genders, and ages. (**E**) UALCAN and LOGpc-based MTHFD1L prognostic significance in UCEC patients of different cancer stages, races, genders, and ages. *P<0.05.

Via OS analysis using KM plotter and LOGpc databases, as shown in Figure 8, MTHFD1L up-regulation was also found to be associated with reduced OS of the BLCA, HNSC, KIRP, LUAD, and UCEC patients of different clinicopathological features including different cancer stages, races, genders and ages, however, most of the correlations were insignificant due to the small sample size used in the analysis. All in all, MTHFD1L overexpression is prognostically relevant to BLCA, HNSC, KIRP, LUAD, and UCEC patients regardless of different clinicopathological features, and can be a promising marker gene for predicting their OS, however, additional experiments are required to be done before clinical application.

### Validating MTHFD1L overexpression using new cohorts

**Figure 9:**
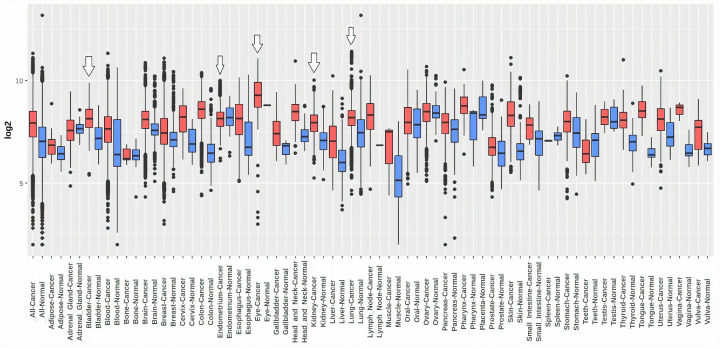
GENT2-based validation of MTHFD1L at transcription level in new BLCA, HNSC, KIRP, LUAD, and UCEC cohorts Blue color represents the normal samples, while red color indicates the cancer samples. A *P*-value (<0.05) was considered as statistically significant.

We re-analyzed the MTDHFD1L expression using new cohorts of BLCA, HNSC, KIRP, LUAD, and UCEC via GENT2 platform. Results of the analysis were in agreement with the previous results and also further verified the significant (*P*>0.05) overexpression of MTHFD1L in BLCA, HNSC, KIRP, LUAD, and UCEC patients relative to controls (Figure 9). Information on the BLCA, HNSC, KIRP, LUAD, and UCEC datasets utilized in this analysis is given in Table 4.

**Table 4:** Information from the BLCA, HNSC, KIRP, LUAD, and UCEC datasets used for the validation of MTHFD1L expression via GENT2

Sr. No.	Cancer	Datasets	Source
1	BLCA	GSE2109, GSE31684, GSE7476, GSE7476, GSE30522, GSE31189, GSE43346, GSE7307, GSE11783, and GSE30522	Affymetrix U133A and U133 Plus2 microarray platforms
2	HNSC	GSE6791, GSE6791, GSE10300, GSE29330, GSE3292, GSE31287, and GSE29330	
3	KIRP	GSE12606, GSE2109, GSE46699, GSE47352, GSE53224, GSE53757, GSE46699, GSE7023, GSE68629, GSE7392, GSE8271, GSE11024, GSE11151, GSE12090, GSE19982, GSE22541, GSE36895, GSE46699, GSE53757, GSE7023, and GSE7307	
4	LUAD	GSE10445, GSE40791, GSE37745, GSE2109, GSE43346, GSE43580, GSE50081, GSE30219, GSE63074, GSE64766, GSE77803, GSE19188, GSE27262, GSE33532, GSE40791, GSE5058, and GSE7307	
5	UCEC	GSE7307, GSE2109, GSE19959, GSE4888, GSE6364, and GSE7307	

### Protein expression level validation of MTHFD1L in BLCA, HNSC, KIRP, LUAD, and UCEC

**Figure 10:**
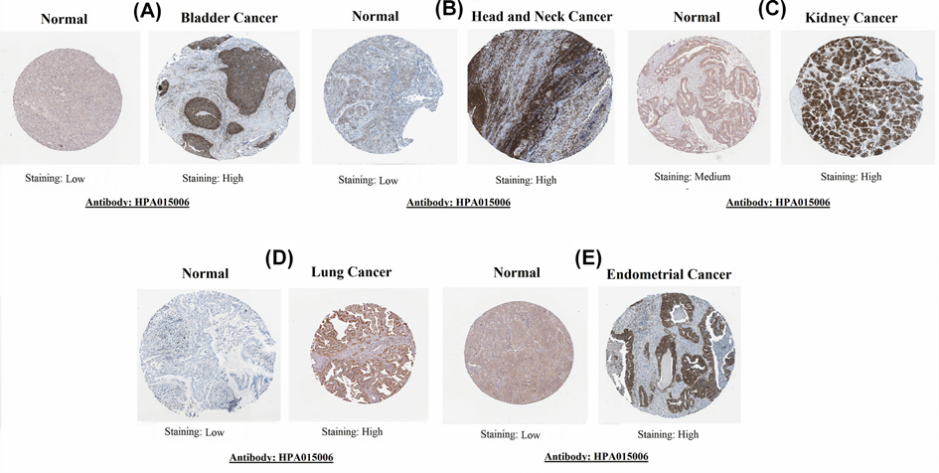
HPA-based validation of MTHFD1L at translational level (×200) (**A**) In bladder cancer, (**B**) in head and neck cancer, (**C**) in kidney cancer, (**D**) in lung cancer, and **(E**) in endometrial cancer.

Next, for protein level expression validation of MTHFD1L, we analyzed IHC results of MTHFD1L through the HPA database. The obtained immunohistochemical images have shown the significant (*P*>0.05) higher expression of MTHFD1L protein in BLCA, HNSC, KIRP, LUAD, and UCEC specimens relative to the normal bladder, head and neck, kidney, lung, and endometrial tissues, which have shown either low (in case of bladder, head and neck, lung, and endometrial tissues) or medium expression (in case of normal kidney tissues) of MTHFD1L (Figure 10). Collectively, our results confirmed that MTHFD1L also overexpressed at the protein level in BLCA, HNSC, KIRP, LUAD, and UCEC as compared with the normal controls.

### Exploring promoter methylation of MTHFD1L

**Figure 11:**
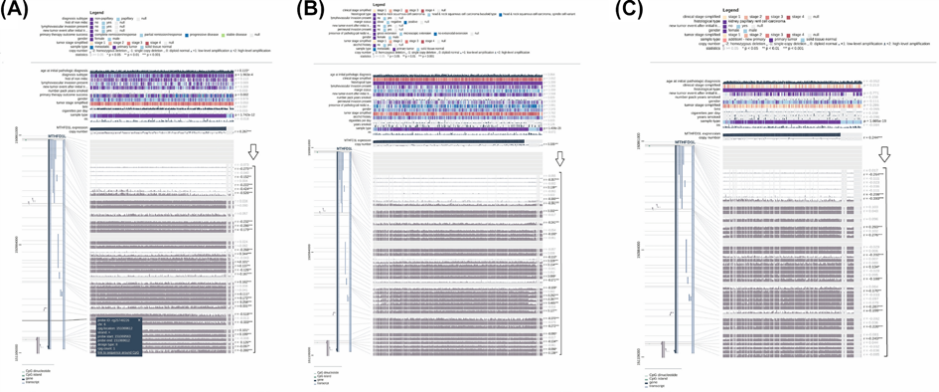
Evaluation of correlation between expression and promoter methylation level of MTFD1L in BLCA, HNSC, and KIRP (**A**) In BLCA, (**B**) in HNSC, and (**C**) in KIRP. A negative sign indicates the negative correlation between MTHFD1L expression and its promoter methylation using a specific probe at a specific CpG island. A *P*-value (<0.05) was considered as statistically significant.

**Figure 12:**
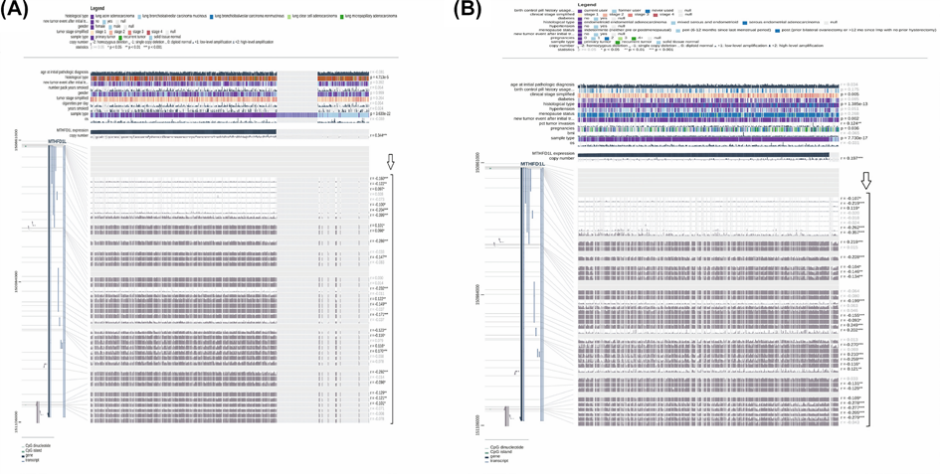
Evaluation of correlation between expression and promoter methylation level of MTFD1L in LUAD and UCEC (**A**) In LUAD and (**B**) in UCEC. A negative sign indicates the negative correlation between MTHFD1L expression and its promoter methylation using a specific probe at a specific CpG island. A *P*-value (<0.05) was considered as statistically significant.

It is earlier known that the dysregulation of different tumor suppressor genes due to aberrant methylation of promoter regions leads to cancer development [27]. To find whether promoter methylation has any impact on MTHFD1L expression or not in BLCA, HNSC, KIRP, LUAD, and UCEC, we have analyzed the Pearson correlation between its promoter methylation and mRNA expression level in BLCA, HNSC, KIRP, LUAD, and UCEC using MEXPRESS. In view of our results, the mRNA expression level of MTHFDL1 was significantly (*P*>0.05) negatively correlated with its promoter methylation (Figures 11 and 12). Taken together, these results highlighted that promoter hypomethylation can affect the expression regulation of MTHFDL1 in BLCA, HNSC, KIRP, LUAD, and UCEC significantly.

### Genetic alteration analysis of MTHFD1L in BLCA, HNSC, KIRP, LUAD, and UCEC

**Figure 13:**
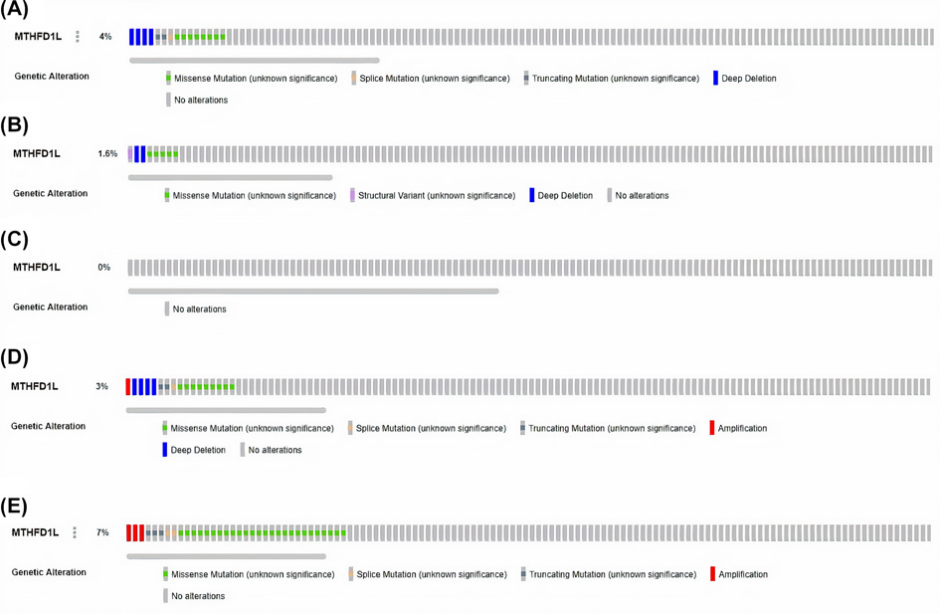
OncoPrint of MTHFDL1 genomic alterations in TCGA BLCA, HNSC, KIRP, LUAD, and UCEC (**A**) In BLCA, (**B**) in HNSC, (**C**) in KIRP, (**D**) in LUAD, and (**E**) in UCEC.

Genetic mutations and CNVs also play major roles in gene expression regulation. We next also explored whether MTHFD1L expression was regulated by these factors or not via cBioportal using TCGA PanCancer Atlas BLCA, HNSC, KIRP, LUAD, and UCEC datasets. No MTHFD1L-associated genetic alterations were observed in KIRP cases, while missense mutations were observed as the most frequent alterations in very small proportions of the analyzed BLCA, HNSC, HNSC, and UCEC cases (Figure 13). Taken together, it is speculated that low percentages of observed genetic alterations in BLCA (4%), HNSC (1.6%), KIRP (0%), LUAD (3%), and UCEC (7%) samples have the least participation in the expression regulation of MTHFD1L.

### Predicted PPI network and pathways of MTHFD1L

**Figure 14:**
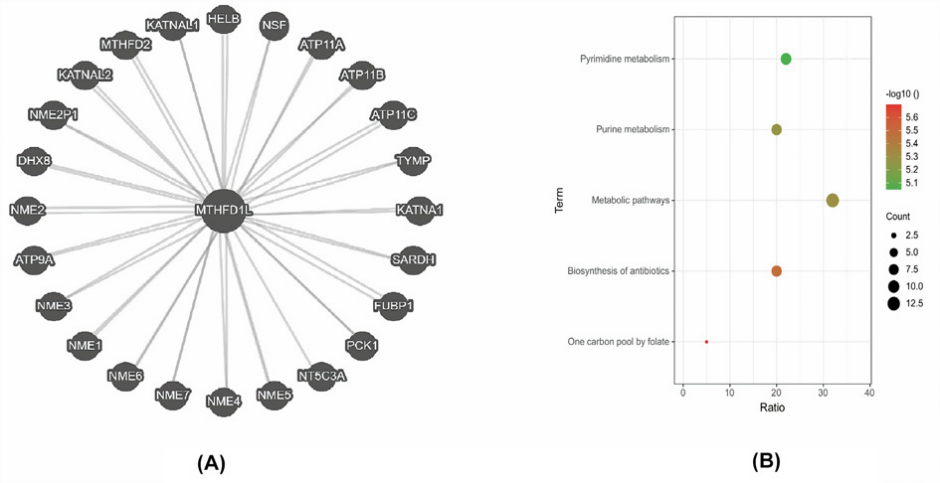
PPI network and KEGG annotation of MTHFD1L-enriched genes (**A**) A PPI network of MTHFD1L-enriched genes and (**B**) KEGG pathway analysis of the MTHFD1L-enriched genes. A *P*-value of <0.05 was considered as significant.

STRING was used in our study to identify the MTHFD1L-enriched genes. The resultant PPI network has highlighted that MTHFD1L was linked with 25 other genes (Figure 13A). All these genes were further subjected to pathway enrichment using the DAVID tool. In view of the analysis results, it was observed that few MTHFD1L-associated genes were significantly (*P*>0.05) involved in five diverse pathways including Pyrimidine metabolism, Purine metabolism, Metabolic Pathways, Biosynthesis of antibiotics, and One carbon pool by folate (Figure 14; Table 5).

**Table 5:** Predicted pathways of MTHFD1L-enriched genes

Pathway	Description	Enriched genes	Gene count	*P*-value
hsa00240	Pyrimidine metabolism	*NT5C3A, NME6, NME7, NME2, NME3, NME4, NME5, TYMP, NME1*	9	<0.05
hsa00230	Purine metabolism	*NT5C3A, NME6, NME7, NME2, NME3, NME4, NME5, NME1*	8	<0.05
hsa01100	Metabolic pathways	*NME2, NME3, NME4, NME5, TYMP, NME1, NT5C3A, MTHFD1L, NME6, NME7, MTHFD2, SARDH, PCK1*	13	<0.05
hsa01130	Biosynthesis of antibiotics	*NME6, NME7, NME2, NME3, NME4, NME5, PCK1, NME1*	8	<0.05
hsa00670	One carbon pool by folate	*MTHFD1L, MTHFD2*	2	<0.05

### MTHFD1L overexpression and infiltrating levels of CD8+ T cells in BLCA, HNSC, KIRP, LUAD, and UCEC patients

**Figure 15:**
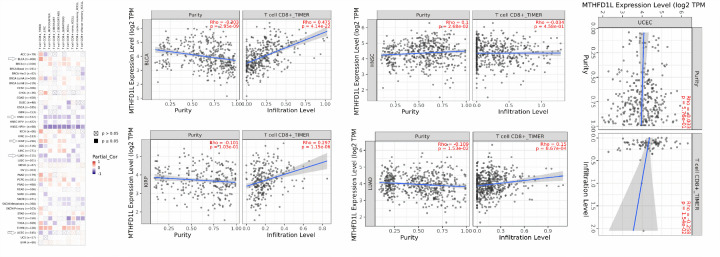
Evaluation of correlations between MTHFD1L expression and CD8+ T immune cells infiltration levels in BLCA, HNSC, KIRP, LUAD, and UCEC via TIMER database A *P*-value (<0.05) was considered as statistically significant.

CD8+ T immune cells play a key role in treating cancer patients through immunotherapy [28]. To further analyze whether the change in MTHFD1L expression exerts any effect on CD8+ T immune infiltration or not, we investigated the correlation of MTHFD1L expression with infiltration levels of CD8+ T immune cells in BLCA, HNSC, KIRP, LUAD, and UCEC using the TIMER database. As a result, we observed that MTHFD1L expression has shown a significant (*P*>0.05) negative correlation with CD8+ T immune cells level in BLCA, HNSC, KIRP, LUAD, and UCEC (Figure 15). Collectively, these findings suggested MTHFDL1 as a possible regulator of CD8+ T immune cells level in BLCA, HNSC, KIRP, LUAD, and UCEC.

### Chemotherapeutic drugs associated with MTHFD1L

**Figure 16:**
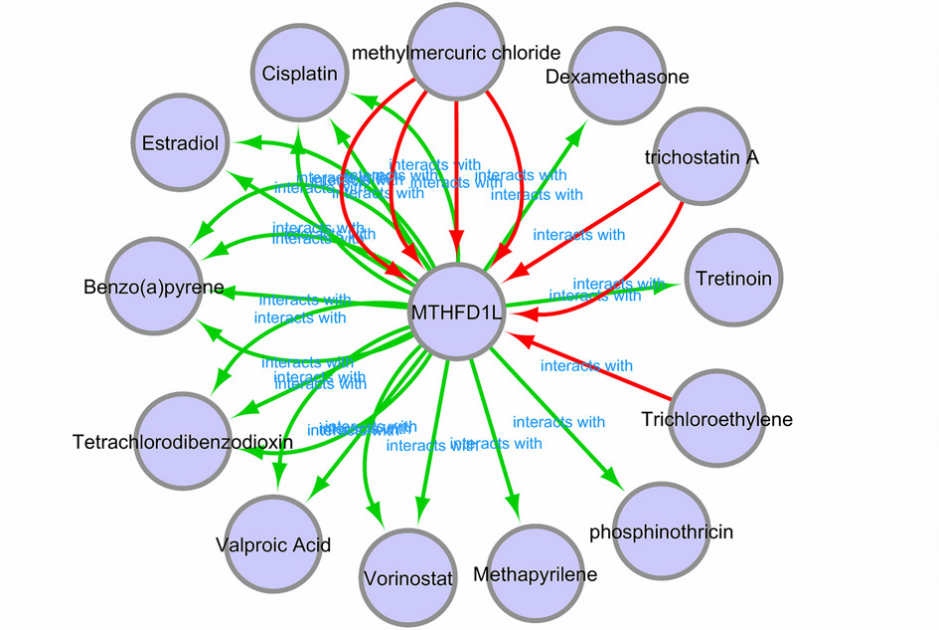
A gene–drug interaction network highlighting MTHFD1L-associated chemotherapeutic drugs Red arrows: chemotherapeutic drugs that can increase the expression of MTHFD1L; green arrows: chemotherapeutic drugs that can suppress the expression of MTHFD1L. A count of arrows between drug and gene in the network represent the supported numbers of studies by the literature.

Different experimentally validated chemotherapeutic drugs associated with MTHFD1L expression were obtained from the CTD database to develop a gene–drug interaction network. Through the constructed network, it was observed that MTHFD1L expression can be regulated by a variety of drugs, such as, cisplatin and vorinostat can up-regulated the expression level of MTHFD1L while tretinon and methylmercuric chloride can suppress MTHFD1L expression level (Figure 16).

## Discussion

Cancer is one of the most killer malignancies in the world that kill millions each year [29]. A lot of literature has confirmed several molecular biomarkers for detection, prediction of prognosis, and treatment of cancer patients [30]. However, still, these biomarkers are not efficient and have various limitations. Thus, there is an urgent need to identify shared biomarkers of cancer that could be used for detection, prediction of prognosis, and treatment of cancer patients without any serious complications.

Earlier studies have reported that MTHFD1L plays a key role in tumor development by dysregulating the folic acid cycle [31,32]. The higher expression of MTHFD1L has been noted in multiple cancers including CRC [9], ESCA [13], and tongue squamous cell carcinoma [14]. In the current study, we explored MTHFD1L expression as a shared potential cancer biomarker in 24 major human cancer subtypes.

We detected the remarkably higher MTHFD1L overexpression in 24 major subtypes of human cancers matched with controls. Also, MTHFD1L overexpression was found to remarkably influence (decrease) the OS duration of BLCA, HNSC, KIRP, LUAD, and UCEC patients only. Collectively, these findings highlighted that MTHFD1L overexpression is involved in the initiation and progression of BLCA, HNSC, KIRP, LUAD, and UCEC; therefore, current research is mainly focusing on these five cancer subtypes. Next, we also re-examined MTHFD1L expression individually in BLCA, HNSC, KIRP, LUAD, and UCEC patients stratified by different clinicopathological parameters. As a result, a significant up-regulation in MTHFD1L expression was also detected in BLCA, HNSC, KIRP, LUAD, and UCEC patients stratified by different cancer stages, races, genders, and ages. Therefore, it is speculated that MTHFD1L might up-regulate in BLCA, HNSC, KIRP, LUAD, and UCEC patients regardless of heterogeneity-barrier.

In terms of promoter methylation impact of MTHFD1L expression, our research has explored the significant negative correlation among expression and promoter methylation levels of the MTHFD1L in BLCA, HNSC, KIRP, LUAD, and UCEC patients. Therefore, ultimately, we speculated that promoter hypomethylation of MTHFD1L may be one of the key factors of its overexpression in BLCA, HNSC, KIRP, LUAD, and UCEC.

Genomic alterations (mutations and CNVs) are known as the key regulator of gene expression dysregulation in cancers [33,34]. Here in this research, we noticed very small percentages of the MTHFD1L genomic abnormalities and CNVs (4, 1.6, 0.3, and 7%) in the analyzed BLCA, HNSC, LUAD, and UCEC samples, respectively. While no MTHFD1L-associated genetic alterations and CNV were found in KIRP. All in all, these findings highlighted that genomic abnormalities have the least involvement in the dysregulation of MTHFD1L, however, further detailed work is required to confirm these results.

Recently, different studies have been conducted worldwide to identify reliable biomarkers for BLCA, HNSC, KIRP, LUAD, and UCEC diagnosis and predict prognosis including significantly dysregulated genes, such as ASPM, CDC20, CENPF, CCNB2, CEP55, KIF20A, NCAPG, HJURP, NUSAP1, TOP2A, TRIP13, TROAP, SPAG5, and TTK in BLCA [35,36]; GPR18, RSPH4A, ULBP2, CNR2, TEX101, CCR8, CCDC39, STC2, MSLN, CHGB, and CNTN5 in HNSC [37,38], LAMC2, CCT3/4/5/6/7/8, and FGFR1-4 in HNSC [39–41]; GATM, MMP1, ARHGEF26, POU2F3, MMP10, PTHLH, and GATA3 in KIRP [42,43]; CDH1, PECAM1, SPP1, IL6, THBS1, SNCA, HGF, CAV1, DLC1, and CDH5 in LUAD [44,45]; TXN, KDM4B, SLC5A1, TXNDC11, COX16, MGAT4A, HSDL2, DAGLA, THRB, PCOLCE2, and ELOVL7 in UCEC [46]. However, to the best of our knowledge, none of these or any other biomarkers have been generalized so far in BLCA, HNSC, KIRP, LUAD, and UCEC patients of different clinicopathological features. Via current research, we have noted the remarkable up-regulation of MTHFD1L in BLCA, HNSC, KIRP, LUAD, and UCEC patients of different clinicopathological features (different cancer stages, patients races, genders, and age groups). Furthermore, MTHFD1L effect on the OS of BLCA, HNSC, KIRP, LUAD, and UCEC patients and its hypomethylation have also confirmed MTHFD1L usefulness as a novel potential biomarker of these cancers. Prior to our knowledge, this research is the first to report a shared biomarker role of MTHFD1L in BLCA, HNSC, KIRP, LUAD, and UCEC, suggesting it as a potential therapeutic candidate in the treatment of cancer.

According to the latest research, the dysregulation of a few important genes may abnormally regulate the tumor microenvironment by altering the immune cells infiltration [47,48]. Keeping this in view, we carried out the correlation analysis among MTHFD1L expression and CD8+ T immune cells infiltration in BLCA, HNSC, KIRP, LUAD, and UCEC. Results highlighted that there is a significant positive correlation between MTHFD1L expression and CD8+ T immune cells level in BLCA and KIRP while a significant negative correlation in HNSC, LUAD, and UCEC. Collectively, this scenario indicates that CD8+ T immune cells may also exert a significant effect on BLCA, HNSC, KIRP, LUAD, and UCEC tumorigenesis via MTHFD1L, however, this role of MTHFD1L ought to be investigated further.

The PPI network of MTHFD1L revealed that it directly interacts with the 25 different genes and a few of them were involved in five diverse pathways including Pyrimidine metabolism, Purine metabolism, Metabolic Pathways, Biosynthesis of antibiotics, and One carbon pool by folate (Table 5). Additionally, we also identified a few potential drugs that could be useful in the treatment of BLCA, HNSC, KIRP, LUAD, and UCEC by regulating the MTHFD1L expression (Figure 16).

## Conclusion

Overexpression of MTHFD1L is associated with tumorigenesis and poor survival in BLCA, HNSC, KIRP, LUAD, and UCEC patients of different clinical parameters. Modulating MTHFD1L expression using different chemotherapeutic drugs may be a promising future treatment option for these cancer patients. However, additional work needed to be done on a large scale prior to clinical implications.

## Data Availability

The datasets analyzed in the current study are available at: http://ualcan.path.uab.edu/cgi-bin/ualcan-res.pl.

## Abbreviations

BLCA: bladder urothelial cancer
CNV: copy number variation
CRC: colorectal cancer
CTD: Comparative Toxicogenomics Database
ESCA: esophageal squamous cell carcinoma
GENT2: Gene Expression across Normal and Tumor tissue
HPA: Human Protein Atlas
IHC: immunohistochemistry
KIRP: kidney renal papillary cell carcinoma
KM: Kaplan–Meier
LUAD: lung adenocarcinoma
MTHFD1L: methylenetetrahydrofolate dehydrogenase 1-like
OS: overall survival
PPI: protein–protein interaction
TCGA: The Cancer Genome Atlas
UCEC: uterine corpus endometrial carcinoma
